# Treatment of Hemorrhage after Extraction of Teeth

**Published:** 1890-02

**Authors:** 


### Treatment of Hemorrhage after Extraction of Teeth.

Mr. Pillin, in a recent issue of the journal of the British Dental Association, relates five cases occurring in his practice of secondary hemorrhage following tooth extraction. Three of the patients were members of the same family, but it is not mentioned whether there was any history of hemorrhagic diathesis. The cases are interesting on account of the length of time elapsing between the operation and the hemorrhage. The first, a man aged twenty-eight had profuse bleeding, commencing on the fifth day; a brother, aged thirty-one, notwithstanding the sockets were plugged immediately after the extraction, on the eighth; and a sister, twenty-five years of age, also on the eighth day. Of the other two cases hemorrhage occurred on the third and fifth days respectively. The treatment adopted successfully was plugging the sockets with cotton-wool saturated with tincture of perchloride of iron, and adapting over this a plate made of Stent's composition, lined with a mixture of tannin and gum tragacanth. Stent's composition is a preparation used by dentists for taking models of the mouth. It is harder than wax at the temperature of the body, and not so flexible as gutta-percha, and therefore makes an accurately fitting and easily applied plug, and can be readily retained in position by keeping the jaws in contact by an ordinary four-tailed bandage.—*Lancet*, Nov. 30, 1889.

